# Propionate represses the *dnaA* gene via the methylcitrate pathway-regulating transcription factor, PrpR, in *Mycobacterium tuberculosis*

**DOI:** 10.1007/s10482-014-0153-0

**Published:** 2014-04-05

**Authors:** Paweł Masiewicz, Marcin Wolański, Anna Brzostek, Jarosław Dziadek, Jolanta Zakrzewska-Czerwińska

**Affiliations:** 1 Department of Microbiology, Ludwik Hirszfeld Institute of Immunology and Experimental Therapy, Polish Academy of Sciences, Wrocław, Poland; 2 Department of Molecular Microbiology, Faculty of Biotechnology, University of Wrocław, Wrocław, Poland; 3 Laboratory of Mycobacterium Genetics and Physiology, Institute of Medical Biology, Polish Academy of Sciences, Łódź, Poland; 4 Structural and Computational Biology Unit, European Molecular Biology Laboratory, Heidelberg, Germany

**Keywords:** PrpR, *dnaA* expression, Propionate, Fatty acids, Tubercle bacillus

## Abstract

During infection of macrophages, *Mycobacterium tuberculosis*, the pathogen that causes tuberculosis, utilizes fatty acids as a major carbon source. However, little is known about the coordination of the central carbon metabolism of *M. tuberculosis* with its chromosomal replication, particularly during infection. A recently characterized transcription factor called PrpR is known to directly regulate the genes involved in fatty acid catabolism by *M. tuberculosis*. Here, we report for the first time that PrpR also regulates the *dnaA* gene, which encodes the DnaA initiator protein responsible for initiating chromosomal replication. Using cell-free systems and intact cells, we demonstrated an interaction between PrpR and the *dnaA* promoter region. Moreover, real-time quantitative reverse-transcription PCR analysis revealed that PrpR acts as a transcriptional repressor of *dnaA* when propionate (a product of odd-chain-length fatty acid catabolism) was used as the sole carbon source. We hypothesize that PrpR may be an important element of the complex regulatory system(s) required for tubercle bacilli to survive within macrophages, presumably coordinating the catabolism of host-derived fatty acids with chromosomal replication.

## Introduction

Faithful transmission of genetic material to daughter cells requires the precise regulation of chromosomal replication and its coordination with the cell cycle. Chromosomal replication in all three domains of life is mainly regulated at the initiation step. In bacteria, it is initiated through cooperative binding of the initiator protein, DnaA, to multiple DnaA boxes (9-mers) within the *oriC* region, which leads to the unwinding of DNA (reviewed in Kaguni 2006; Leonard and Grimwade 2011; Ozaki and Katayama 2009). The activity and availability of both key elements of replication initiation, DnaA and *oriC*, are tightly regulated to ensure that replication is initiated only once per cell cycle. Several factors that regulate replication initiation have been identified in both Gram-negative (*Escherichia coli, Caulobacter crescentus*) and Gram-positive (*Bacillus subtilis, Streptomyces coelicolor*) bacteria (Zakrzewska-Czerwińska et al. 2007; Katayama et al. 2010; Wolański et al. 2012). A few regulatory systems, such as the inactivation of DnaA-ATP by ATP hydrolysis, appear to be used universally by all bacteria. In contrast, others systems appear to be specific for particular bacteria, such as the CtrA, GcrA and CcrM proteins, which temporarily and spatially coordinate replication initiation with cell differentiation and cell cycle progression in *C. crescentus* (Collier 2012).

Surprisingly, little is known about the regulation of DNA replication in response to various environmental conditions and factors (Wang and Levin 2009). Recent studies have demonstrated that there is a direct link between central carbon metabolism (CCM) and the initiation and elongation stages of DNA replication in *E. coli* (Maciąg et al. 2011, 2012) and *B. subtilis* (Jannière et al. 2007). These discoveries indicate the existence of a global correlation between metabolic status and the key cell cycle processes leading to bacterial proliferation (e.g., replication). In this light, it is important to investigate the relationship between CCM and replication in intracellular pathogens such as *Mycobacterium tuberculosis* that utilize compounds “scavenged” from the host.

The success of *M. tuberculosis* as the causative agent of TB lies mostly in its ability to maintain a dormant, non-replicating state for extended periods under unfavorable conditions (reviewed in Gengenbacher and Kaufmann 2012). During the infection of macrophages, *M. tuberculosis* is exposed to nutrient limitation and thus must re-route its carbon metabolism from sugars to fatty acids and cholesterol (see McKinney et al. 2000; Munoz-Elias and McKinney 2006; Shi et al. 2010). The CCM of *M. tuberculosis* is known to be a key determinant of its pathogenicity (Rhee et al. 2011), but little is known about the coordination of CCM with replication during the transition to dormancy.

In the present work, we show for the first time that PrpR, a transcription factor that regulates genes encoding enzymes responsible for fatty acid catabolism (Masiewicz et al. 2012), represses *dnaA* expression during *M.* *tuberculosis* growth on propionate as a sole carbon source.

## Materials and methods

### DNA manipulations, bacterial strains, culture conditions, and protein purification

DNA manipulations were carried out using standard protocols (Sambrook et al. 1989). Enzymes were supplied by Fermentas and Promega; [γ-^32^P]ATP radioisotope was purchased from Hartmann Analytic; and oligonucleotides were synthesized by Genomed (Poland). The utilized bacterial strains and oligonucleotides, as well as their relevant characteristics, are given in Table 1. *M. tuberculosis* strain H37Rv and its derivatives were cultured aerobically at 37 °C in Middlebrook 7H9 broth (Difco, Detroit, MI.) or on 7H10 agar plates supplemented with 10 % OADC (oleic acid–albumin–dextrose–catalase) and 25 μg/ml kanamycin (when required). For RNA extraction and gene expression measurements, *M. tuberculosis* strains were cultivated at 37 °C either in 7H9 + OADC medium or in M9 minimal salts medium (Sambrook et al. 1989), containing 2 mM MgSO_4_ and 0.1 mM CaCl_2_, with glucose, sodium acetate or sodium propionate (0.5 % each) as a sole carbon source. The fusion protein, 6HisPrpR, was purified using affinity chromatography (HIS-Select HF resin), as described previously by Masiewicz et al. (2012).

**Table 1:** Bacterial strains and oligonucleotides (primers)

Bacterial strains		
Strain	Genotype	Source
*M. tuberculosis* H37Rv	Laboratory strain (Cole et al. 1998)	Laboratory stock
*M. tuberculosis* Δ*prpR*	*M. tuberculosis* H37Rv with deleted *prpR* gene (unmarked deletion)	Masiewicz et al. (2012)
*M. tuberculosis* Δ*prpR* + pMV*prpR*	*M. tuberculosis* Δ*prpR*-complemented strain containing a pMV306-integrated functional copy of the *prpR* gene under the control of its own promoter	Masiewicz et al. (2012)

Oligonucleotides (primers)		
Oligonucleotide	Sequence (5′–3′)	Application
MtrpmH_Fw	GGTCCTTTTGCCCTTGGTCAC	Affinity chromatography
biotMtrpmH_Rv	biot-TGAACCGGGGTCATCGGTCAA	
MtoriC_Fw	CGGGATCCCACGGCGTGTTCTTCCGACAACG	
biotMtoriC_Rv	biot-CTCTTGTCGTAGCCGCGTCCAT	
pmtdnaA_Fw2	CCGTTTCAGCGTGGAAACGG	Amplification of *dnaA* promoter region for EMSA and IP
MtrpmH_Rv	TGAACCGGGGTCATCGGTCAA	
p1129_Fw	GACGTCAACCGGATCGGCAGC	Amplification of *prpDR* promoter region for EMSA and IP
p1129_Rv	GGCACCGGAAAACGTCCTCGA	
pmtrA_Fw	CTTGCGGTCTCTGCCGAGCTC	Amplification of *mtrA* promoter region for EMSA and IP
pmtrA_Rv	TGGTGTCCATGGTGTCACCACA	
RT_MtdnaA_Fw	TACACGCGGCAGGCAACT	*dnaA* (*rv0001*) transcription
RT_MtdnaA_Rv	GGAGACATATTTGACCCGCATT	
RT_1129_Fw	GAGGGATACCGTTCATCTTCGT	*prpR* (*rv1129c*) transcription
RT_1129_Rv	GCGGACTGTCGCTTTGAGA	
RT_MtsigA_Fw	GGTGATTTCGTCTGGGATGAA	*sigA* (*rv2703*) transcription
RT_MtsigA_Rv	GCTACCTTGCCGATCTGTTTG	

### Affinity chromatography

The intergenic *rpmH*–*dnaA* DNA fragment (645 bp) and the *oriC* region (557 bp) were PCR amplified using *M. tuberculosis* strain H37Rv chromosomal DNA as a template and two primer pairs: the MtrpmH_Fw primer plus the 5′-biotin-labeled reverse primer, MtrpmH_Rv; and the MtoriC_Fw primer plus the 5′-biotin-labeled MtoriC_Rv primer (Table 1). The resulting biotinylated DNA fragments (10 pmol) were immobilized on Streptavidin Magnetic Dynabeads (Dynabeads^®^ M-280 Streptavidin, Invitrogen). For experiments, *M. tuberculosis* cultures were grown on 7H9 + OADC broth to an OD_600_ of 0.7–0.9. The cells were harvested by centrifugation and resuspended in chilled phosphate buffered saline (PBS; 0.8 % NaCl, 0.02 % KCl, 0.144 % Na_2_HPO_4_ and 0.024 % KH_2_PO_4_) supplemented with 1 mM EDTA and protease inhibitors (Complete Protease Inhibitor Cocktail Tablets, Roche). The cells were disrupted in the presence of silica beads (BioSpec Products) using a Mini-BeadBeater-8 (BioSpec Products) for 1 min. The samples were then centrifuged (16,000×*g*, 15 min, 4 °C) and the protein concentrations in the supernatants were determined using the Bradford assay (Bradford 1976). For “fishing” experiments, 5 mg of total protein extract (final volume, 1 ml) was incubated with 10 pmol of DNA-immobilized Dynabeads with constant gentle mixing for 1 h at 25 °C. The Dynabeads were then washed and eluted with PBS supplemented with increasing NaCl concentrations. The eluates were resolved by 10 % SDS-PAGE, and the gels were stained with Coomassie brilliant blue. Visible protein band was excised from the gel and analyzed by mass spectrometry.

### Whole-cell immunoprecipitation assay

The immunoprecipitation assay was performed as described previously (Jakimowicz et al. 2007; Masiewicz et al. 2012). Briefly, *M. tuberculosis* H37Rv wild type and *prpR*-deletion strains were cultivated in 7H9 + OADC broth at 37 °C to OD_600_ = 0.9 and treated with glutaraldehyde (1 %) for 5 min, and the cross-linked nucleoprotein complexes were immunoprecipitated with anti-PrpR antibodies, as described previously (Masiewicz et al. 2012). Cells treated in parallel without cross-linking served as negative controls. The immunoprecipitated DNA was PCR amplified using primers flanking the *dnaA* and *prpR* promoter regions. Two independent experiments of immunoprecipitation assays were carried out.

### Electrophoretic mobility shift assay (EMSA)

EMSAs were carried out as described previously (Masiewicz et al. 2012). A DNA fragment (100–150 ng) was incubated with increasing amounts of purified 6HisPrpR protein in 1× binding buffer (50 mM Tris–HCl pH 7.5, 50 mM KCl, 10 mM MgCl_2_, 10 % glycerol, 0.5 mM EDTA) for 30 min at 25 °C. The nucleoprotein complexes were resolved on 4 % polyacrylamide gels at approximately 8 °C in 0.25× TBE buffer at 5–10 V cm^−1^, and complexes were analyzed with a Typhoon 8600 Variable Mode Imager and the Image Quant software (Molecular Dynamics).

### RNA extraction and reverse transcription

RNA was extracted from *M. tuberculosis* cultures incubated in M9 minimal medium containing various carbon sources. Since this minimal medium does not sustain robust growth of *M. tuberculosis*, bacteria were first precultured in 7H9 + OADC broth to an OD_600_ of 0.6–0.8. Next, cells were washed twice with M9 minimal medium containing acetate, glucose or propionate (0.5 %), resuspended in the respective minimal medium and incubated at 37 °C for an additional 48 h. The cultures were then collected by centrifugation (6000×*g*, 10 min, 4 °C), and RNA extraction was carried out according to the previously described protocol (Masiewicz et al. 2012). The reverse transcription reactions were carried out using a SuperScript III First-Strand Synthesis SuperMix kit (Invitrogen) and random hexanucleotides, according to the manufacturer’s protocol. The reaction products were analyzed by agarose gel electrophoresis.

### Quantitative real-time PCR

SYBR green-based real-time PCR (2× HS-PCR Mix SYBR A; A&A Biotechnology) was used to quantify the mRNA levels of the *dnaA* gene in *M. tuberculosis* H37Rv wild type, Δ*prpR*, and complemented strains cultivated on media with different carbon sources. To exclude the possibility of secondary structure formation, we used the Primer Express 3.0 (Applied Biosystems) application to design the primers, which are listed in Table 1. Reactions were performed on a StepOne Plus apparatus with the StepOne Software 2.0 (both from Applied Biosystems), according to the manufacturer’s instructions. The relative quantity of *dnaA* mRNA was determined by reference to the mRNA levels of the *M.* *tuberculosis* housekeeping gene, *sigA*. Three independent experiments with cDNA templates derived from RNA isolated from three independent *M. tuberculosis* cultures were carried out.

### Statistical analysis

Differences between experimental groups were determined by the Student’s *t* test. *P* values of <0.05 were considered significant, and were calculated using the Excel 2007 software (Microsoft Office).

## Results and discussion

### PrpR interacts weakly with the *dnaA* promoter region

**Fig. 1:**
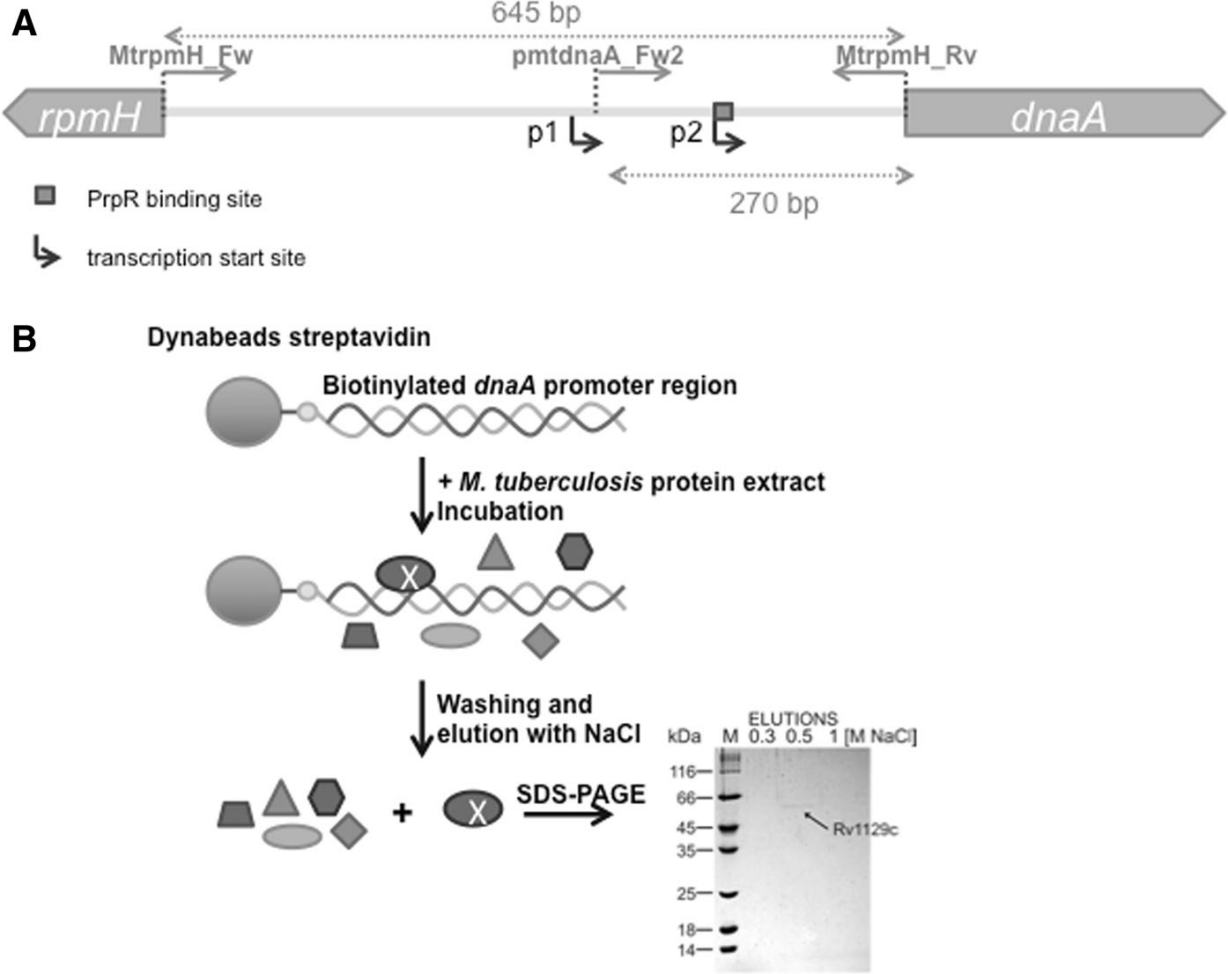
Identification of protein(s) binding the *rpmH*–*dnaA* intergenic region of *M. tuberculosis.* **A** Schematic depiction of the *M. tuberculosis rpmH*–*dnaA* intergenic region. The positions of transcription start sites, p1, p2 (Salazar et al. 2003; Li et al. 2010) and PrpR binding site are indicated by *bend arrow* and *gray square*, respectively. Primers MtrpmH_Fwor, pmtdnaA_Fw2, and MtrpmH_Rv were used to amplify a 645-bp intergenic region for affinity chromatography or a 270-bp *dnaA* promoter fragment for EMSA analysis (Table 1). **B** Streptavidin affinity chromatography using a biotinylated *rpmH*–*dnaA* intergenic region as bait. The PCR amplified *rpmH*–*dnaA* region (10 pmol) was immobilized on Dynabeads and incubated with protein extracts (5 mg) prepared from *M. tuberculosis* H37Rv grown on 7H9 + OADC medium (OD_600_ = 0.9). The Dynabeads were then washed and eluted with PBS buffer supplemented with increasing concentrations of NaCl. The eluted proteins were resolved by 10 % SDS-PAGE, and visible bands were excised for analysis by mass spectrometry

**Fig. 2:**
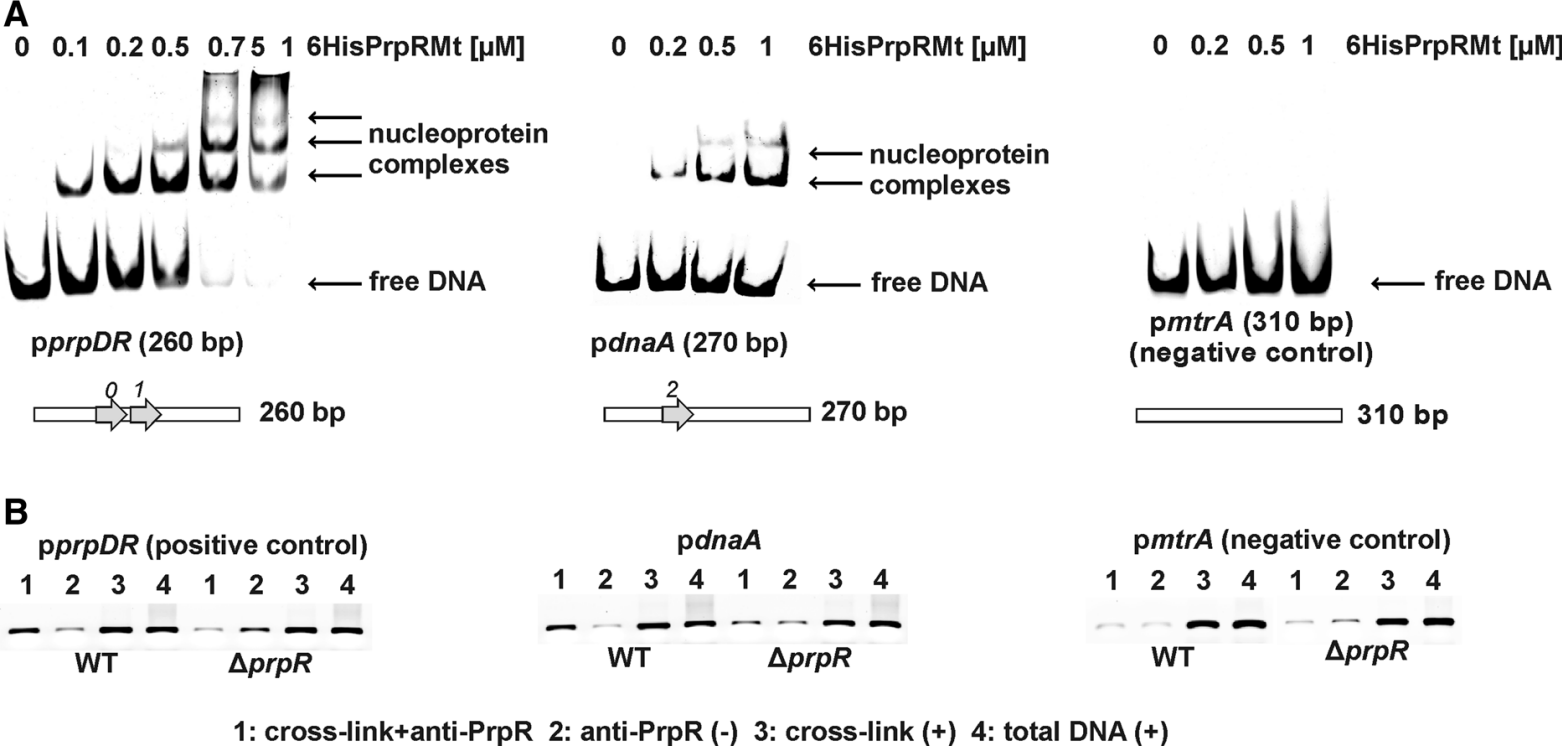
PrpR interacts with the *dnaA* promoter region in vitro and in vivo. **A** EMSA: a DNA fragment containing the *dnaA* promoter region (PCR amplified with primers pmtdnaA_Fw2 and MtrpmH_Rv, Table 1) was incubated with a non-specific competitor and increasing amounts of 6HisPrpR, and the nucleoprotein complexes were analyzed on a 4 % polyacrylamide gel; p*rpDR* (containing the perfect PrpR biding site) and the *mtrA* promoter region served as positive and negative controls, respectively. *Arrows* along horizontally orientated* bars* inserted below each figure indicate number of PrpR boxes in corresponding DNA fragment. Digits above arrows indicate number of nucleotide mismatches from the consensus (TTTGCAAA) within each PrpR box. **B** In vivo immunoprecipitation: PrpR–DNA complexes cross-linked with glutaraldehyde were immunoprecipitated with anti-6HisPrpR polyclonal antibodies (sample 1). PCR was carried out with the following primer pairs: p1129_Fw and p1129_Rv (p*prpDR,* positive control); pmtdnaA_Fw2 and MtrpmH_Rv (p*dnaA*); and pmtrA_Fw and pmtrA_Rv (p*mtrA*, negative control). A second negative control (2) consisted of extracted DNA subjected to immunoprecipitation without cross-linking. Positives controls (+) were also performed using templates obtained from strains subjected only to cross-linking (3) or total DNA extracted from the cells (4)

To identify *dnaA* promoter (p*dnaA*)- and *oriC* region-binding protein(s) that might control the initiation of replication in *M. tuberculosis*, we performed streptavidin affinity chromatography using biotinylated DNA fragment (Fig. 1b) containing either the *dnaA* promoter region (645 bp, Fig. 1a) or the *oriC* region (557 bp, data not shown) as bait against cell extracts prepared from *M. tuberculosis* H37Rv cultured to an OD_600_ of 0.9 on 7H9 + OADC medium. We identified a putative *dnaA* promoter region-binding protein of ~60 kDa (Fig. 1b) but no binding protein was identified for the *oriC* region (presumably due to the fact that frequently the interactions between proteins and *oriC* are transient). The protein was identified by mass spectrometry (47 % protein sequence coverage) as corresponding to Rv1129c (PrpR), a recently described transcriptional factor that is directly involved in regulating the methylcitrate and glyoxylate pathways (Masiewicz et al. 2012). The PrpR protein was shown to bind the promoter regions of genes encoding the key enzymes of the methylcitrate (methylcitrate dehydratase [PrpD] and methylcitrate synthase [PrpC]) and glyoxylate (isocitrate lyase [Icl1]) cycles. In addition, we recently showed that PrpR interacts specifically with the 8-bp palindromic sequence, TTTGCAAA (Masiewicz et al. 2012). In the present work, using in silico search we identified only one putative PrpR binding sequence in the *dnaA* promoter; this sequence differs from the high affinity binding sequence by two nucleotides (TTTtCAAc). Interestingly, the identified sequence is located immediately downstream the transcription start site (transcription from the p2*dnaA* promoter, see Fig. 1a) (Salazar et al. 2003; Li et al. 2010). To verify that PrpR was able to bind the *dnaA* promoter region in vitro, we performed an EMSA. This binding was confirmed, although the purified PrpR protein exhibited a lower affinity toward the *dnaA* promoter region than toward a DNA fragment containing the prefect palindrome (e.g., its own promoter, p*prpR*) (Fig. 2a). A single nucleoprotein PrpR–*pdnaA* complex was visible in reactions containing 200 nM protein, whereas incubation with higher protein concentrations (500, 1,000 nM) resulted in the formation of two nucleoprotein complexes probably as a result of PrpR dimer binding (PrpR forms stable dimers and possibly trimers, Masiewicz et al. 2012). It has to be noted that the *mtrA* promoter region, which served as a negative control, was not bound by the PrpR protein (even at highest concentration). Surface plasmon resonance (SPR) analysis confirmed the weak interaction between PrpR and the *dnaA* promoter. The dissociation of PrpR from the *dnaA* promoter was much faster than that from the *prpR* promoter region, suggesting that the complexes between PrpR the *dnaA* promoter are not stable (data not shown).

To determine whether the PrpR regulator binds the *dnaA* gene promoter region in *M. tuberculosis,* we performed glutaraldehyde cross-linking of proteins to DNA in intact cells, followed by selective immunoprecipitation of the protein–DNA complexes with anti PrpR antibodies, as described previously (Masiewicz et al. 2012). Both PrpR–p*dnaA* and PrpR–p*prpR* (positive control) complexes were detected, whereas no signal (or faint background signal) was observed from a *prpR*-deletion strain or PrpR–p*mtrA*, which served as negative controls (Fig. 2b).

Collectively, these results indicate that PrpR binds the *dnaA* promoter region relatively weakly in vitro and within intact *M.* *tuberculosis* cells. This suggests that, in addition to the regulation of fatty acid catabolism, PrpR might play a role in regulating the expression of the *dnaA* gene, which is responsible for the initiation of chromosomal replication.

### PrpR represses transcription of *dnaA* during growth on propionate

**Fig. 3:**
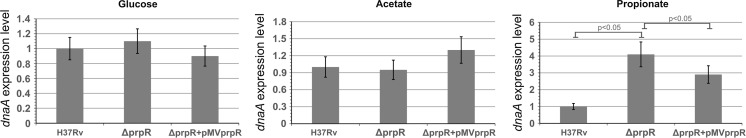
PrpR represses *dnaA* gene expression during growth on propionate. Total RNA was extracted from cultures grown in M9 minimal medium containing glucose, acetate or propionate (0.5 %) as the carbon source. PCR was carried out with primers RT_MtdnaA_Fw and RT_MtdnaA_Rv. The mRNA levels of *dnaA* were normalized with respect to that of the constitutively expressed housekeeping gene, *sigA*. Means were calculated from three independent experiments and three determinations per experiment. The *error bars* indicate standard deviations of triplicate samples. Statistical significance was calculated by the Student’s *t* test

We previously demonstrated that the *prpR* gene is most highly expressed during the growth of *M. tuberculosis* on propionate, a degradation product of odd-chain-length fatty acids and cholesterol (Masiewicz et al. 2012). The growth of *M. tuberculosis* on medium containing propioniate as the sole carbon source was impaired in contrast to growth on glucose (Masiewicz et al. 2012). Under such conditions, one would expect that the high expression of PrpR might influence the expression of the *dnaA* gene. To test this hypothesis, we performed qRT-PCR analysis of *dnaA* expression using RNA isolated from the wild type and *prpR*-deleted *M. tuberculosis* strains growing on M9 minimal medium containing glucose, acetate or propionate as a sole source of carbon. In this experiment, we measured the activity of both p1 and p2 *dnaA* promoters (using primers complementary to *dnaA* gene, Table 1). The expression level of *dnaA* in the Δ*prpR* strain growing on propionate was four-fold higher than that in the wild type strain, suggesting that PrpR may act as a repressor of *dnaA* gene expression (Fig. 3). Notably, we did not observe any significant difference in *dnaA* expression when these strains were cultivated on minimal medium containing glucose or acetate (Fig. 3). It must be mentioned that in the *M. tuberculosis* Δ*prpR* complemented strain the repression of *dnaA* gene on propionate was not completely restored (Fig. 3) (despite the fact that the expression level of *prpR* gene was not altered compared to the wild type strain, data not shown). However, the complementing copy of the *prpR* gene was introduced on the *M. tuberculosis* chromosome together with its promoter region containing strong (TTTGCAAA) PrpR boxes (in addition to the PrpR binding sites present in the native locus—a common *prpD* and *prpR* promoter region), which presumably titrated PrpR protein away from the *dnaA* promoter region what could result in higher that in the wild type *dnaA* expression. It is noteworthy that the expression of *prpD* gene (activated by PrpR) in the *M. tuberculosis ΔprpR* complemented strain did also not reach the level measured in the wild type strain (Masiewicz et al. 2012).

**Fig. 4:**
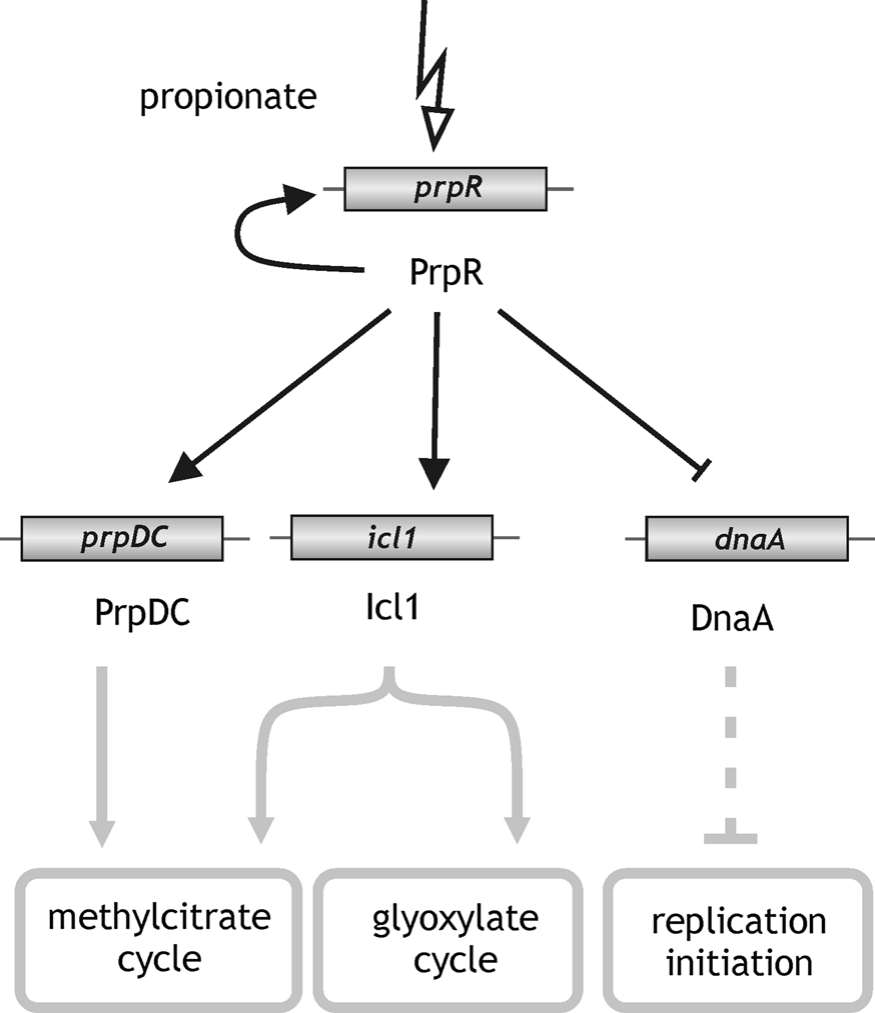
PrpR coordinates fatty acid catabolism with the initiation of chromosomal replication. In *M. tuberculosis*, PrpR activates the expression of genes involved in the methylcitrate and glyoxylate cycles, and also inhibits *dnaA* gene expression*. Perpendicular lines* indicate negative regulation. Adapted from Masiewicz et al. (2012)

The relatively low affinity of PrpR towards the *dnaA* promoter is presumably compensated by the elevated level of the protein in *M. tuberculosis* during growth on propionate. It is worth mentioning that *M. tuberculosis* utilizes fatty acids and cholesterol as a major carbon source in host macrophages during infection (Gengenbacher and Kaufmann 2012; Rhee et al. 2011). Thus, the data presented here and in our earlier study (Masiewicz et al. 2012) suggest that PrpR might play a dual role in media containing fatty acids and cholesterol as the sole carbon source, acting both as an activator of genes involved in fatty acid catabolism including its own gene and as a repressor of the *dnaA* gene (Fig. 4). It is worth mentioning that besides PrpR there are also DnaA and MtrA proteins that may be directly involved in negative regulation of *dnaA* gene expression in *Mycobacterium* by binding *dnaA* promoter region (Salazar et al. 2003; Fol et al. 2006; Nguyen et al. 2010). It is noteworthy to point out that the MtrA is supposed also to negatively influence DnaA oligomerization along *oriC* and thus chromosome replication by binding DnaA boxes within *oriC* (Rajagoplan et al. 2010).

Tubercle bacilli can survive for decades in humans or hypoxic- and nutrient-depleted media (Hett and Rubin 2008). However, surprisingly little is known about the regulatory mechanisms responsible for the ability of these bacilli to enter and exit dormancy. Based on the novel findings presented herein and on our earlier study (Masiewicz et al. 2012) we hypothesize that PrpR could be an important element of the complex regulatory system(s) required for the persistence of TB within macrophages, controlling both the catabolism of host-derived fatty acids and the initiation of chromosomal replication (Fig. 4).
